# Risk Factors Associated with Default from Multi- and Extensively Drug-Resistant Tuberculosis Treatment, Uzbekistan: A Retrospective Cohort Analysis

**DOI:** 10.1371/journal.pone.0078364

**Published:** 2013-11-06

**Authors:** Maeve K. Lalor, Jane Greig, Sholpan Allamuratova, Sandy Althomsons, Zinaida Tigay, Atadjan Khaemraev, Kai Braker, Oleksander Telnov, Philipp du Cros

**Affiliations:** 1 Médecins Sans Frontières, Nukus, Karakalpakstan, Uzbekistan; 2 Médecins Sans Frontières, London, United Kingdom; 3 Ministry of Health, Karakalpakstan, Uzbekistan; 4 Médecins Sans Frontières, Berlin, Germany; University of California, San Francisco, United States of America

## Abstract

**Background:**

The Médecins Sans Frontières project of Uzbekistan has provided multidrug-resistant tuberculosis treatment in the Karakalpakstan region since 2003. Rates of default from treatment have been high, despite psychosocial support, increasing particularly since programme scale-up in 2007. We aimed to determine factors associated with default in multi- and extensively drug-resistant tuberculosis patients who started treatment between 2003 and 2008 and thus had finished approximately 2 years of treatment by the end of 2010.

**Methods:**

A retrospective cohort analysis of multi- and extensively drug-resistant tuberculosis patients enrolled in treatment between 2003 and 2008 compared baseline demographic characteristics and possible risk factors for default. Default was defined as missing ≥60 consecutive days of treatment (all drugs). Data were routinely collected during treatment and entered in a database. Potential risk factors for default were assessed in univariate analysis using chi-square test and in multivariate analysis with logistic regression.

**Results:**

20% (142/710) of patients defaulted after a median of 6 months treatment (IQR 2.6–9.9). Factors associated with default included severity of resistance patterns (pre-extensively drug-resistant/extensively drug-resistant tuberculosis adjusted odds ratio 0.52, 95%CI: 0.31–0.86), previous default (2.38, 1.09–5.24) and age >45 years (1.77, 1.10–2.87). The default rate was 14% (42/294) for patients enrolled 2003–2006 and 24% (100/416) for 2007–2008 enrolments (p = 0.001).

**Conclusions:**

Default from treatment was high and increased with programme scale-up. It is essential to ensure scale-up of treatment is accompanied with scale-up of staff and patient support. A successful first course of tuberculosis treatment is important; patients who had previously defaulted were at increased risk of default and death. The protective effect of severe resistance profiles suggests that understanding disease severity or fear may motivate against default. Targeted health education and support for at-risk patients after 5 months of treatment when many begin to feel better may decrease default.

## Introduction

Multidrug-resistant tuberculosis (MDR-TB) is defined as *Mycobacterium tuberculosis* resistant to at least isoniazid and rifampicin, the two most effective anti-TB drugs. In 2011, an estimated 630,000 of the world’s 12 million prevalent cases of TB were MDR, and an estimated 310,000 (range 220,000–400,000) of these were among notified TB patients with pulmonary TB [1], [2]. The highest proportions of MDR-TB are found in the countries of Eastern Europe and Central Asia. In Uzbekistan a TB drug-resistance survey completed in 2011 found that 23% of new and 62% of previously treated TB patients had MDR-TB [1]. Uzbekistan is one of the 27 WHO high-burden MDR-TB countries and was recently ranked highest globally in terms of estimated incidence of newly transmitted MDR-TB within the population [1], [3]. Treatment for MDR-TB uses second-line drugs that are less effective than those used for first-line treatment and which often have significant side-effects [4]. It is also lengthy, complex and expensive [5]. As a result, treatment outcomes for patients with MDR-TB are worse than for drug-sensitive TB, with low success rates and high rates of treatment failure and default [4]. While some analyses of MDR-TB treatment cohorts have reported factors associated with negative outcomes of treatment (death, failure and default) [6], [7], [8], it is not clear that the risk factors associated with these three outcomes are necessarily the same.

Factors associated with default for drug-sensitive TB programmes are well described and include: individual patient profile (e.g. illicit drug use), clinical status or therapy (e.g. side-effects of drugs) or health-service characteristics (e.g. distance of patients from clinic or the way that patients are treated by staff) [9]. Factors associated with default from MDR-TB have been less well described.

Médecins Sans Frontières (MSF), together with the Ministry of Health, initiated a DOTS-plus project to treat MDR-TB in 2003 in Karakalpakstan, Uzbekistan. The initial programme model and patients’ outcomes have been described [10]. By June 2011, 1796 patients with TB showing any form of drug resistance had been enrolled in treatment in Karakalpakstan. Despite psychosocial support being offered to patients, rates of default from treatment have been relatively high, increasing particularly since 2007 when the programme was scaled up. In a retrospective cohort analysis, we aimed to determine factors associated with default in MDR and extensively drug-resistant (XDR) TB patients who started treatment between 2003 and 2008 and thus had the opportunity to finish 2 years of treatment by the end of 2010.

## Methods

### Study Setting

The Republic of Karakalpakstan is a sovereign republic in the north-west of the Republic of Uzbekistan. Although it covers approximately one-third of the country of Uzbekistan, it contains less than 6% of the population, around 1.6 million people. The prevalence of TB in Karakalpakstan in 2009 was reported as 433/100,000 population, considerably higher than in the rest of Uzbekistan (227/100,000) [11], [12]. From 2003, MSF in collaboration with the Ministry of Health enrolled patients from Nukus city and Chimbay district in a DOTS-plus programme.

### Definitions

Registration groups based on patients’ treatment history were assigned according to WHO standards; a patient with a TB treatment history of more than 1 month at the time of sputum submission was considered to be previously treated [13]. Treatment outcomes were defined according to WHO definitions, and were determined by a medical team including Ministry of Health and MSF doctors. Successful treatment outcomes included “cure” and “treatment complete”. Default was defined as missing at least 60 days of consecutive doses of all drugs. Pre-XDR TB was defined as MDR-TB with resistance to an injectable anti-TB drug (capreomycin or kanamycin) or a fluoroquinolone (ofloxacin), but not both. XDR-TB was defined as MDR-TB with resistance to both an injectable anti-TB drug (capreomycin or kanamycin) and a fluoroquinolone.

### Study Population and Programme Characteristics

Drug susceptibility testing (DST) was initially provided only for patients with suspected drug-resistant TB, but was expanded to include all smear-positive patients from 2006. A total of 830 patients with confirmed drug-resistant TB were started on treatment between October 2003 and 31^st^ December 2008 in two districts of Karakalpakstan: 21 had mono-resistant (2.5%), 94 polydrug-resistant (11.5%), 710 MDR (85.5%) - 140 of whom were pre-XDR - and four XDR (0.5%) TB. Analysis for this study was restricted to 710 patients with MDR or XDR-TB, excluding four patients who transferred in or out of the programme. Programme characteristics from 2003–2005 have been described [10]. All patients were hospitalised at the commencement of treatment; as the programme scaled up, criteria for discharge were altered to allow earlier discharge for ongoing ambulatory treatment. Individualised treatment regimens were updated during the study period to reflect updated international guidelines [13]. Health educators were replaced by psychosocial support counsellors in 2007 with the aim of maximising adherence. Food was provided in hospital, and monthly food packages were provided for discharged patients. Financial support was provided for patient transport to outpatient facilities (until 2009) and incentives were paid to Ministry of Health doctors and nurses (until 2009).

### Laboratory Testing

Sputum smear microscopy, culture and DST were conducted according to international standards in the Nukus mycobacteriology laboratory. Smears were assessed using Ziehl-Neelsen staining or auramine fluorescence microscopy and culture using Lowenstein-Jensen Media. DSTs were performed on Lowenstein-Jensen cultures in the Nukus mycobacterial laboratory and repeated at the supranational reference laboratory in Borstel, Germany, for samples until April 2006, as previously described [14]. Thereafter, external quality control tests of the Nukus laboratory were evaluated by the Borstel laboratory and by Gauting supranational reference laboratory in Germany from 2009. The Nukus mycobacterial laboratory was found to have successfully passed the external quality assessment of drug susceptibility testing each year to date. The BACTEC™ MGIT™ 960 liquid culture system (BD, Franklin Lakes, NJ, USA) was introduced to the laboratory for first-line drug testing in September 2007. First-line DSTs were performed using the critical concentration method in the BACTEC™ MGIT™ 960 system using BACTEC™ MGIT™ 960 SIRE Kit and the BACTEC™ MGIT™ 960 PZA Kit (BD, Franklin Lakes, NJ, USA). Drug susceptibility testing for second-line antibiotics was performed on Lowenstein-Jensen media by the proportion method.

### Data Collection

Data were routinely collected from patient forms and registers over the course of treatment and entered into a custom-made Epi-Info project database (Epi-Info version 6, CDC, Atlanta, USA). Data collected at baseline included demographics, prior history of TB treatment, most recent DST results and regimen. Follow-up data included adherence, outcome and laboratory results. Data were exported into STATA (version 10.1, StataCorp LP, Texas, USA) for analysis.

### Statistical Analyses

A retrospective cohort analysis was performed on baseline demographic characteristics and possible factors associated with default in M/XDR TB patients enrolled for treatment between 2003 and 2008. Proportions were compared between defaulters and successfully treated patients using the chi-square test for demographic characteristics (sex, age, marital status) and possible risk factors for default (unemployed, body-mass index [BMI] <18 kg/m^2^, use of alcohol or tobacco, previous imprisonment, previous travel outside region, previous TB treatment, previous default from TB treatment and resistance profile at diagnosis). Referent groups for resistance profile at diagnosis were as follows: resistance to 2^nd^ line drugs compared to resistance to 1^st^ line drugs only, pre-XDR/XDR compared to MDR with no fluorquinolone or injectable resistance, resistant to ≥5 drugs compared to resistant to <5 drugs. Factors that were associated with default in univariate analysis at p<0.10 were assessed in multivariate analysis using a logistic regression model with stepwise forward selection, including a variable for the time period (2007 or later) when a number of programmatic characteristics changed as treatment was scaled up. Likelihood ratio tests were conducted after each stepwise addition to assess which variables to retain in the model. Similar regression analyses were performed for the outcomes of death and failure.

The study met the standards set by the MSF Ethics Review Board for retrospective analyses of routinely collected programmatic data.

## Results

Among 710 M/XDR-TB patients enrolled in the DOTS-plus programme between 2003 and 2008∶62% (438) were successfully treated according to WHO definitions, 20% (142) defaulted from treatment, 7% (50) died and 11% (80) failed treatment. The median age of patients was 29.4 years (IQR 23.1–40.3) and 48% (343) were male. The length of time patients were hospitalised decreased in 2007 to a median of 3.7 months and 3.4 months in 2008, compared to 5.0–9.5 months in each of the previous 4 years. The median overall time on treatment was 20 months. Possible risk factors were assessed for patients who were successfully treated compared with those who defaulted from treatment. For patients enrolled between 2003 and 2006 the proportion who defaulted was 14.3% (42/294); this increased significantly to 24% (100/416) for patients enrolled in 2007 and 2008 (p = 0.001). The median time to last dose of drugs taken by defaulters was 6.1 months from the start of treatment (IQR 2.6–9.9), with 59.9% (85/142) defaulting in the ambulatory phase of treatment. While approximately 25% of defaulters took their last dose in autumn or summer, fewer did so in winter (20/142, 14%, p<0.001) and more did so in spring (48/142, 34%, p = 0.03).

Factors associated with default from treatment (univariate analysis) included patients who were 45 years or older, had previously travelled outside the region, or had previously defaulted from treatment. Protective factors for default included previous failure of category II treatment (defined as an 8-month retreatment regimen with first-line drugs), previous treatment with second-line drugs and a more severe resistance profile (ie pre-XDR/XDR resistance) at diagnosis (Table 1).

**Table 1:** Baseline proportions and association of factors with default by univariate analysis.

		Success N (%)	Default N (%)	p
**Total patients unless otherwise stated**		**438**	**142**	
**Demographic** **characteristics**	Sex (male)	207 (47.3)	79 (55.6)	0.083
	Age (>45 years)	68 (15.5)	**34 (23.9)**	**0.022**
	BMI <18 kg/m^2^	202 (46.0)	70 (49.3)	0.510
	Married	223 (50.9)	68 (47.9)	0.531
	Unemployed	211 (48.2)	74 (52.1)	0.415
**Potential high** **risk groups**	Tobacco use	55 (12.6)	21 (14.8)	0.493
	Alcohol use	63 (14.4)	27 (19.0)	0.185
	Prisoner (at/prior to admission)	22 (5.0)	12 (8.5)	0.131
	Travel outside Karakalpakstan	71 (16.2)	**35 (24.7)**	**0.024**
	MDR Contact	27 (6.2)	8 (5.6)	0.818
**Registration group/** **Previous TB treatment**	New	74 (16.9)	20 (14.10)	0.430
	Relapse	124 (28.3)	47 (33.1)	0.277
	Treatment after default	20 (4.6)	**13 (9.2)**	**0.040**
	Treatment after failing cat I	32 (7.3)	13 (9.2)	0.474
	Treatment after failing cat II	**119 (27.2)**	25 (17.6)	**0.022**
	Other	69 (15.8)	24 (16.9)	0.746
	Previous second-line treatment	**171 (39.0)**	42 (29.6)	**0.042**
**Resistance profile** **at diagnosis**	Resistant to second-line drugs(compared to first-line drugs only)	153/437 (35.0)	43/142 (30.3)	0.301
	Pre-XDR/XDR (compared to MDR with nofluoroquinolone or injectable resistance)	**117/437 (26.7)**	23/142 (16.2)	**0.011**
	Resistant to ≥5 drugs (compared toresistant to <5 drugs)	192/437 (43.9)	54/142 (38.0)	0.216

BMI = body-mass index. MDR = multidrug-resistant. Cat I = category I treatment. Cat II = category II treatment. XDR = extensively drug-resistant.

After adjustment through multivariate analysis, patients with more severe resistance patterns were protected from default (pre-XDR/XDR compared to less resistance (MDR with no fluoroquinolone or injectable resistance) adjusted odds ratio [aOR] = 0.52; 95% CI 0.31–0.86; p = 0.011), while patients who had previously defaulted from treatment (aOR = 2.38; 95% CI 1.09–5.24; p = 0.030), older patients (aOR = 1.77; 95% CI 1.10–2.87; p = 0.020) and those whose treatment started in 2007 or 2008 (aOR = 1.70; 95% CI 1.10–2.73; p = 0.030) were more likely to default (Table 2).

**Table 2:** Factors associated with default identified by multivariate analysis.

	Univariate analysis		Multivariate analysis	
	OR (95% CI)	p	OR (95% CI)	p
Pre-XDR/XDR	0.53 (0.32–0.87)	**0.011**	0.52 (0.31–0.86)	**0.011**
Age (>45 years)	1.71 (1.07–2.73)	**0.022**	1.77 (1.10–2.87)	**0.020**
Treatmentafter default	2.10 (1.02–4.37)	**0.040**	2.38 (1.09–5.24)	**0.030**
Time period(2007 or later)	1.71 (1.13–2.58)	**0.010**	1.70 (1.10–2.73)	**0.030**
Treatment afterfailing cat II	0.57 (0.35–0.93)	**0.022**	0.85 (0.49–1.49)	0.585
Travel outsideKarakalpakstan	1.69 (1.07–2.68)	**0.023**	1.43 (0.87–2.35)	0.157
Sex (male)	1.40 (0.96–2.05)	0.083	1.25 (0.83–1.89)	0.278

OR = odds ratio. XDR = extensively drug-resistant. Cat II = category II treatment.

Factors associated with death in multivariate analysis were low BMI (<18 kg/m^2^) at admission (aOR = 3.5; 95% CI 1.7–7.3; p = 0.001), more than one cavity at diagnosis (aOR = 3.4; 95% CI 1.3–9.1; p = 0.014) and treatment after default from previous treatment (aOR = 3.0; 95% CI 1.2–7.6; p = 0.020). No factors associated with treatment failure on univariate analysis remained significant after adjustment for co-factors in multivariate analysis.

## Discussion

The main factors associated with default in our cohort after adjustment in multivariate analysis were age (>45 years), previous default from tuberculosis treatment and less severe DST profile at diagnosis. In a retrospective analysis of a cohort in Latvia, no identified factors were found to be significantly associated with default from MDR-TB treatment [8]. An analysis of patients treated in MDR-TB programmes in Estonia, Latvia, Philippines, Russia and Peru between 2000–2004 reported primarily socio-economic factors as predictors for default, but did not discuss whether this varied by country [15]. Factors identified in other countries as associated with default included alcohol or drug use [16], [17], substandard housing, increased number of drugs for treatment, centralised treatment [18], homelessness, unemployment, history of imprisonment, and baseline positive smear result [15]. Our results thus highlight the need for MDR-TB programmes to identify local risk factors for default.

As the programme was scaled up, default rates rose in our cohort from 14% to 24%, concurrent with increased patient numbers. Our rates are not atypical; a meta-analysis of over 9000 patients reported an overall default rate of 23% [19]. Such high rates show the urgent need to better understand predictive factors for default from MDR-TB treatment.

In contrast to default from treatment, factors associated with death were largely related to severity of disease at initiation of treatment. The association between default and re-treatment after previously defaulting (aOR 2.38) shows the importance of success with the first regimen of TB treatment offered to a patient. Patients who had previously defaulted were at increased risk of not only subsequent default, but also of death. Previously treated patients should have additional support at the start of a new treatment to emphasise the importance of adherence and completion. Before starting a new course of treatment, patients who previously defaulted need special attention to ascertain and address the issues that contributed to them defaulting.

Interestingly, patients with more severe resistance profiles (pre-XDR/XDR) were less likely to default, perhaps indicating that an understanding of the severity of disease or fear were motivations to persevere with treatment. This finding contrasts with an analysis of patients in the Philippines, who were at increased risk of default with increasing numbers of drugs in their regimen [18]. We hypothesise that many patients with less severe resistance profiles feel better after 6 months of treatment and may believe they are cured, reducing the perceived value of continuing treatment, whereas patients with more severe resistance profiles may be more aware of the gravity of their disease and thus are less likely to default. More exploration of these hypotheses is needed and qualitative interviews are planned. Targeted health education of these patients after 5 months of treatment, when many patients have begun to feel better, may decrease default from treatment.

As the programme was scaled up, default rates increased. During the period of analysis, programmatic changes were made in order to scale-up access to drug-resistant TB diagnosis and treatment, resulting in about 200 patients with drug-resistant TB starting treatment annually from 2007 onwards. These changes included an increase in the ratio of patients starting treatment per doctor/nurse treating, a decrease in the length of hospitalisation, a decrease in the size of food packages given to patients in ambulatory treatment, a change in drug regimen (initial treatment changed from ethionamide to prothionamide), and a change in patient profile (more patients without previous TB treatment were included in later years). These factors have not been included in our review of baseline factors as they aligned too closely with the period before and after scale-up, and thus their potential role can only be hypothesised. Similar results to ours were found in a meta-analysis of default in drug-resistant TB with rates tending to increase with treatment cohort size [20]. If the WHO policy to scale-up to universal access for drug-resistant TB diagnosis and treatment [2] is to be successful, it is essential to ensure that scale-up of patient numbers is matched by scale-up of staff, decentralising patient treatment in order to spread patient burden across sites and increasing patient support.

While many factors contribute to default from MDR-TB treatment, our results suggest that overall TB treatment outcomes could be improved with strengthened education during the first TB treatment, and targeted educational and psychosocial support to those at greatest risk of default from MDR-TB treatment. These interventions should support patients in the challenges they face, help them understand the severity of the disease and motivate by emphasising both the value of treatment availability and the risks of defaulting from treatment.

Further work is required to investigate the reasons why the factors we identified are associated with increased risk of default and how they can be addressed. The limitations of our study include lack of testing for susceptibility to amikacin, meaning that some pre-XDR or XDR patients may have been misclassified as MDR. However, cross-resistance between kanamycin and amikacin is high in the programme region [21] so misclassification is unlikely to have affected many patients. Other limitations include that the study analysed retrospective data and that we reviewed only baseline factors, which mean that we could not assess the effects of changes in the programme over time or other factors which could influence default such as patient, treatment and service factors. The strengths of the study include the large cohort size and thus the large number of defaulters available for analysis.

Default rates from MDR-TB treatment are known to be high and defaulters have an increased risk of mortality, resistance amplification and transmission of resistant TB within the community. It is important for programmes to identify local risk factors for default and for further research to demonstrate the best programme models for reducing default. Current ambitions to achieve universal access to treatment for drug-resistant TB [2] will fail to curb the spread of disease if default rates are not addressed.
